# Synchronized Survey Scan Approach Allows for Efficient Discrimination of Isomeric and Isobaric Compounds during LC-MS/MS Analyses

**DOI:** 10.1155/2018/2046709

**Published:** 2018-04-01

**Authors:** Keabetswe Masike, Ntakadzeni Madala

**Affiliations:** Department of Biochemistry, University of Johannesburg, P.O. Box 524, Auckland Park 2006, South Africa

## Abstract

Liquid chromatography-mass spectrometry- (LC-MS-) based multiple reaction monitoring (MRM) methods have been used to detect and quantify metabolites for years. These approaches rely on the monitoring of various fragmentation pathways of multiple precursors and the subsequent corresponding product ions. However, MRM methods are incapable of confidently discriminating between isomeric and isobaric molecules and, as such, the development of methods capable of overcoming this challenge has become imperative. Due to increasing scanning rates of recent MS instruments, it is now possible to operate MS instruments both in the static and dynamic modes. One such method is known as synchronized survey scan (SSS), which is capable of acquiring a product ion scan (PIS) during MRM analysis. The current study shows, for the first time, the use of SSS-based PIS approach as a feasible identification feature of MRM. To achieve the above, five positional isomers of dicaffeoylquinic acids (diCQAs) were studied with the aid of SSS-based PIS method. Here, the MRM transitions were automatically optimized using a 3,5-diCQA isomer by monitoring fragmentation transitions common to all five isomers. Using the mixture of these isomers, fragmentation spectra of the five isomers achieved with SSS-based PIS were used to identify each isomer based on previously published hierarchical fragmentation keys. The optimized method was also used to detect and distinguish between diCQA components found in *Bidens pilosa* and their isobaric counterparts found in *Moringa oleifera* plants. Thus, the method was shown to distinguish (by differences in fragmentation patterns) between diCQA and their isobars, caffeoylquinic acid (CQA) glycosides. In conclusion, SSS allowed the detection and discrimination of isomeric and isobaric compounds in a single chromatographic run by producing a PIS spectrum, triggered in the automatic MS/MS synchronized survey scan mode.

## 1. Introduction

Plants produce a myriad of organic compounds referred to as secondary metabolites (natural products) which differ in their structure and biosynthetic origins. These metabolites undergo chemical modifications, such as conjugation [1, 2], and isomerization (positional and geometrical) [3–6], which further contribute to the high complexity of the plant metabolome. For instance, secondary metabolites such as hydroxycinnamic acid (HCA) derivatives have the potential to form conjugates with organic acids such as isocitric acid [1], tartaric acid [2, 7–9], and quinic acid [4, 10, 11], thus forming hydroxycinnamoyl-isocitric acid [1], hydroxycinnamoyl-tartaric acid [2, 7–9], and hydroxycinnamoyl-quinic acid [4, 10, 11], respectively. The most common HCA derivatives include caffeic acid, ferulic acid, and *p*-coumaric acid, to name a few.

In addition, these HCA derivatives undergo isomerization to produce positional isomers such as di-acylated hydroxycinnamoyl-quinic acid derivatives like 1,3-dicaffeoylquinic acid (1,3-diCQA) or 1,5-diCQA [3–6]. As such, some HCA conjugates have been found to result in isobaric compounds which produce similar mass spectrometry (MS) fragmentation patterns [1], and this renders identification challenging from an analytical perspective. For instance, diCQA positional isomers, apart from being isobaric constituents of each other, also produce similar fragmentation patterns to structurally related molecules such as caffeoylquinic acid (CQA) glycosides [12–14], making identification in different plant species very challenging.

From the above, it can be surmised that secondary metabolites are diverse and, in some cases, are unique to specific plant species and, as such, can be used as chemotaxonomic markers [15–18]. Therefore, the unambiguous detection and identification of these metabolites using analytical techniques such as liquid chromatography-mass spectrometry (LC-MS) is of paramount importance. For targeted analysis, multiple reaction monitoring (MRM) using a triple quadrupole LC-MS/MS system can be employed to selectively screen for and detect and quantify metabolites of interest [19–22]. However, studies dedicated to providing specific fragmentation patterns that discriminate between isomeric and isobaric secondary metabolites during MRM analyses are limited [23, 24]. As such, a novel method/approach referred to as synchronized survey scan (SSS) is proposed as a possible way of discriminating structurally similar metabolites during MRM analyses, especially when they produce similar fragmentation transitions. Using a SSS function, we have demonstrated that positional isomers of diCQAs and their isobaric compounds, CQA glycosides, can be distinguished in a single chromatographic run. Here, authentic standards and plant extracts of *Bidens pilosa* [25] and *Moringa oleifera* were employed, since these plant species are reported to, respectively, accumulate/produce these compounds. Thus, the overall aim of the current study was to use the SSS approach as an orthogonal identification component of the MRM method for efficient discrimination of structurally related plant metabolites.

## 2. Materials and Methods

### 2.1. Materials

Authentic standards (with the purity of above 99.6%) of dicaffeoylquinic acids (1,3-diCQA, 1,5-diCQA, 3,4-diCQA, 3,5-diCQA, and 4,5-diCQA) were purchased from Phytolab (Vestenbergsgreuth, Germany). Mass spectrometry grade (99.9%) methanol was purchased from Romil Pure Chemistry (Cambridge, UK). Mass spectrometry grade formic acid (with the purity of above 96%) was obtained from Sigma-Aldrich (St. Louis, MO, USA). The analytical column used was a reverse-phase Raptor biphenyl (2.1 × 100 mm, 3 *µ*m) column purchased from Restek (Bellefonte, PA, USA).

### 2.2. Methods

#### 2.2.1. Sample Preparation

A 1 mg/mL solution for each diCQA positional isomer was prepared with 100% methanol. The solution (for each positional isomer) was diluted 10× with 100% methanol. Furthermore, equal amounts (e.g., 40 *µ*L) were taken from each positional isomer sample to prepare a mixed sample (e.g., of a final volume of 200 *µ*L). The samples (individuals and the mixture) were placed in amber vials and subjected to HPLC-PDA analyses.

#### 2.2.2. Metabolite Extraction

The dried leaves of *B. pilosa* and *M. oleifera* were pulverized, respectively, using a clean and dry quartz mortar and pestle. Extraction was conducted using an organic solvent-based extraction. The respective amounts of samples (0.2 g) were mixed with 2 mL of 50% aqueous methanol, and these extracts were placed (with the lids of the tubes closed to avoid evaporation) in a heating block at 60°C for 2 h. The samples were sonicated for 30 min using an ultrasonic bath and then centrifuged at 9740 ×*g* for 10 min at 4°C. The resulting supernatants were subjected to UHPLC-MS analyses.

#### 2.2.3. Ultra-High Performance Liquid Chromatography Tandem Mass Spectrometry (UHPLC-MS/MS) Analysis

Once prepared, samples were analyzed on a Shimadzu Nexera 8050 UHPLC (Kyoto, Japan) fitted with a Raptor biphenyl analytical column, with the column temperature set at 40°C. A binary solvent mixture consisting of MilliQ water made up of 0.1% formic acid (eluent A) and methanol made up of 0.1% formic acid (eluent B) at a constant flow rate of 0.4 mL/min was used to analyze 2 *µ*L of the injected samples. For the gradient elution, the following conditions were used: isocratic 5% eluent B from 0 min to 1 min, linear 5%–20% eluent B from 1 min to 5 min, linear 20%–90% eluent B from 5 min to 40 min, and isocratic 90% eluent B from 40 min to 45 min. At the end of analysis, conditions were changed to the initial conditions (5% eluent B) from 45 min to 48 min and finally, the column was reequilibrated with isocratic 5% eluent B from 48 min to 52 min. The data were acquired using a UV detector set at 325 nm and 330 nm.

For the MS analysis, the chromatographic effluent was introduced to a MS source and ionized by electrospray (ESI). ESI conditions were as follows: the interface voltage was set at 3.0 kV (in the negative ESI mode), the source temperature was 300°C, nitrogen was used as the drying gas at the flow rate of 10.00 L/min, and as a nebulizing gas at a flow rate of 3.00 L/min. Argon was used as a collision gas with a pressure of ±230 kPa in the collision cell. Sensitive and qualitative analysis of isomeric and isobaric plant metabolites was achieved by developing a MRM and MRM-dependent product ion scan (PIS) method. The MRM transitions were developed or optimized using 3,5-diCQA as the sample of choice based on the work done by Clifford et al. [3]. According to Clifford and colleagues, 3,5-diCQA contains product ions (e.g., *m/z* 353 representing a caffeoylquinic acid moiety, *m/z* 179 representing a caffeic acid moiety, and *m/z* 191 representing a quinic acid moiety) characteristic of all diCQA isomers, as well as CQA glycosides [13, 14]. The MRM transition parameters were automatically optimized to produce the transitions shown in Table 1. The dwell time for all the MRM transitions was 30 ms.

A synchronized survey scan (SSS) function was selected which automatically performed MS/MS analysis when the precursor threshold peak intensity exceeded 2,000,000. This resulted in a combined MRM and a MRM-dependent PIS, both of which were produced in a single analysis. For the PIS mode, ions were collected at a mass range 100–1000 Da with a continuous scan time of 1 sec, at a collision energy of 25 eV.

## 3. Results and Discussion

### 3.1. LC-MS/MS Method Optimization

**Figure 1:**
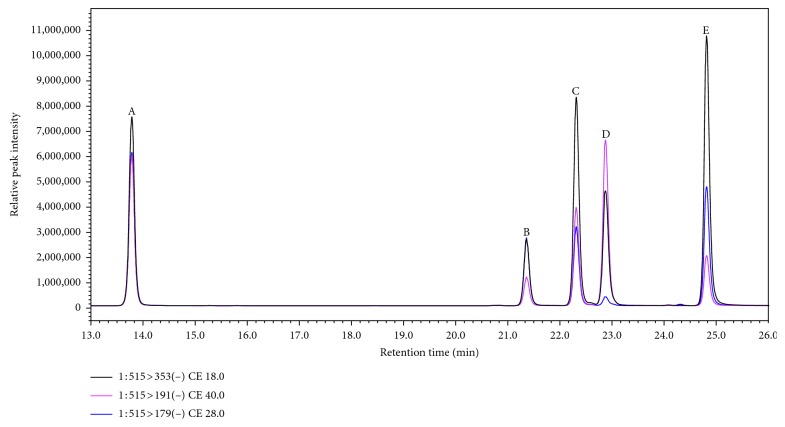
Chromatogram showing differences in the relative abundance of the MRM transitions of a sample containing a mixture of dicaffoylquinic acids (diCQAs) authentic standards: **A** = 1,3-diCQA, **B** = 3,4-diCQA, **C** = 3,5-diCQA, **D** = 1,5-diCQA, and **E** = 4,5-diCQA.

**Figure 2:**
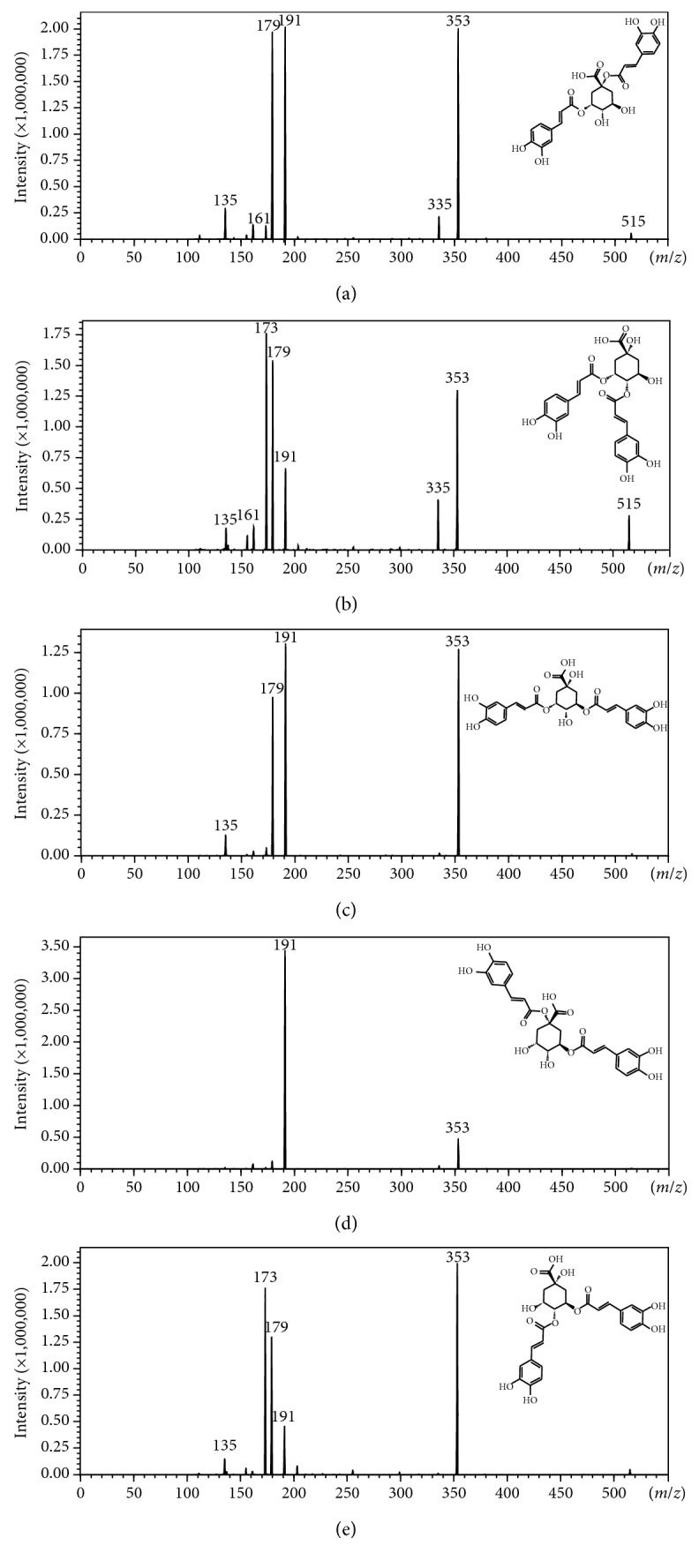
Product ion scan (PIS) spectra and fragmentation pathways of (a) 1,3-diCQA, (b) 3,4-diCQA, (c) 3,5-diCQA, (d) 1,5-diCQA, and (e) 4,5-diCQA authentic standards.

**Figure 3:**
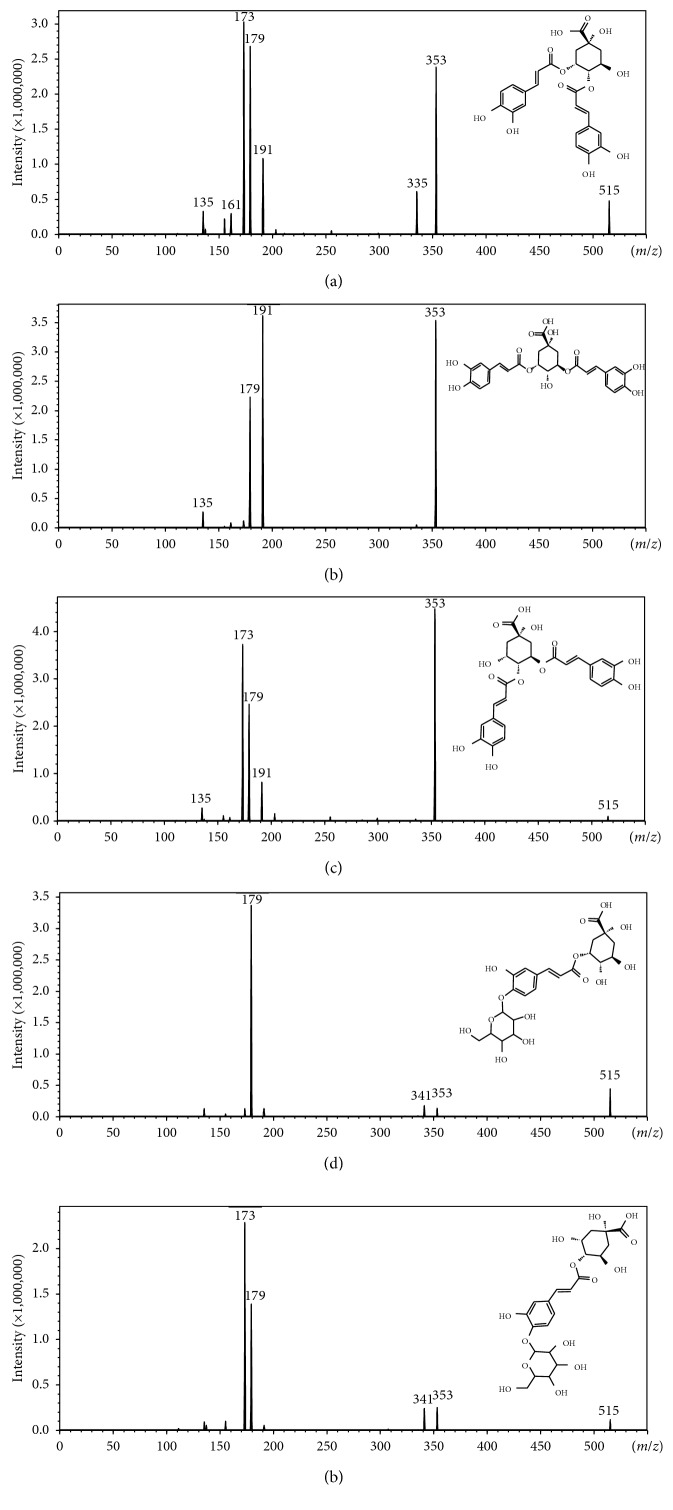
Product ion spectra and fragmentation pathway of diCQA positional isomers: (a) 3,4-diCQA, (b) 3,5-diCQA, and (c) 4,5-diCQA detected in *Bidens pilosa* plant extracts. Product ion spectra and fragmentation pathway of CQA glycoside positional isomers: (d) 3-*O*-(4′-*O*-caffeoyl glucosyl) quinic acid and (e) 4-*O*-(4′-*O*-caffeoyl glucosyl) quinic acid detected in *Moringa oleifera* plant extracts.

In this study, LC-MS analysis was used to sensitively and qualitatively analyze isomeric and isobaric plant metabolites by developing a MRM-dependent product ion scan (PIS) method. Samples (authentic standards and plant samples) containing positional isomers of dicaffeoylquinic acids (diCQAs) and caffeoylquinic acid (CQA) glycosides were analyzed under reverse-phase chromatographic conditions using a Raptor biphenyl column with methanol as part of the binary solvent mixture. To identify the respective diCQA positional isomers, a sample made up of a mixture of the positional isomers (authentic standards) was analyzed, and it produced a chromatogram showing well-resolved peaks representing the five isomers (Figure 1; Table 2). The retention times (Rts) of the respective peaks were compared with the Rts of the individual (nonmixed) authentic standards, and the elution order under the abovementioned conditions was noted as 1,3-diCQA (Figure 1, **A**), 3,4-diCQA (Figure 1, **B**), 3,5-diCQA (Figure 1, **C**), 1,5-diCQA (Figure 1, **D**), and 4,5-diCQA (Figure 1, **E**). Furthermore, the respective product ion scan (PIS) spectra, triggered in the automatic MS/MS synchronized survey scan mode, were also referred to for the analysis of the fragmentation patterns of the diCQA positional isomers for further identification (Figure 2). Although the MRM transition parameters were automatically optimized using 3,5-diCQA, all five diCQA isomers were detected, since these compounds have been shown to share similar product ions [3, 11].

### 3.2. MRM and MRM-Dependent Product Ion Scan Analysis of Plant Extracts

After optimization, *Bidens pilosa* and *Moringa oleifera* plant extracts were analyzed under the abovementioned conditions (Section 3.1). When the MRM transition parameters, which were automatically optimized using 3,5-diCQA, were used to analyze extracts of *B. pilosa* and *M. oleifera* plant samples, several peaks were detected. Briefly, three peaks were detected in *B. pilosa*, and only two peaks were detected in *M. oleifera*. When compared, the two peaks detected from *M. oleifera* samples showed an earlier elution profile (Rt = 4.89 and 7.32) and were thus observed to be more hydrophilic than the three peaks detected in *B. pilosa* samples, which showed a later elution profile (Rt = 21.24, 22.05, and 24.46). These observations are summarized in Table 2. Such chromatographic observations are important in scenarios whereby the analyzed plant sample contains both diCQAs and CQA glycosides, thus circumstances whereby the plant extract contains both the isomeric and isobaric compounds [11, 13, 14, 26].

The Rts of the peaks (Rt = 21.29, 22.05, and 24.61) found in *B. pilosa* samples were compared with those observed with the diCQA authentic standard samples, and the respective peaks were identified as 3,4-diCQA (Figure 3(a)), 3,5-diCQA (Figure 3(b)), and 4,5-diCQA (Figure 3(c)) (Figure 3; Table 2), since the fragmentation pattern of these peaks was identical to the fragmentation pattern observed in Figures 2(a), 2(c) and 2(e), respectively. For the peak representing 3,4-diCQA, the observed fragment ions were (Figure 3(a)) at *m/z* 353 ([caffeoylquinic acid-H]^−^) due to the loss of a caffeic acid moiety [3], at *m/z* 335 ([caffeoylquinic acid-H_2_O-H]^−^) due to the dehydration of the caffeoylquinic acid moiety which subsequently results in a lactone group on the quinic acid [27], at *m/z* 191 ([quinic acid-H]^−^) as a result of a loss of a caffeic acid moiety from a caffeoylquinic acid group, at *m/z* 179 ([caffeic acid-H]^−^) ascribed to the loss of the caffeoylquinic acid moiety (or the loss of a quinic acid moiety from a caffeoylquinic acid group), at *m/z* 173 ([quinic acid-H_2_O-H]^−^) (base peak, bp) due to the dehydration of a quinic acid moiety, at *m/z* 161 ([caffeoylquinic acid-quinic acid moiety-H_2_O-2H]^−^) as a result of a loss of a dehydrated quinic acid moiety from a caffeoylquinic acid lactone, and at *m/z* 135 ([caffeic acid-CO_2_-H]^−^) due to the decarboxylation of a caffeic acid moiety (Figures 2(b) and 3(a); Table 2). The peak representing 3,5-diCQA showed fragment ions (Figure 3(b)) at *m/z* 353 ([caffeoylquinic acid-H]^−^), 191 ([quinic acid-H]^−^) (bp), 179 ([caffeic acid-H]^−^), and 135 ([caffeic acid-CO_2_-H]^−^). Lastly, the peak identified as 4,5-diCQA showed product ions (Figure 3(c)) at *m/z* 353 ([caffeoylquinic acid-H]^−^) (bp), 191 ([quinic acid-H]^−^), 179 ([caffeic acid-H]^−^), and 173 ([quinic acid-H_2_O-H]^−^). It is worth noting that in the absence of authentic standards, the ion at *m/z* 173 is diagnostic of HCA derivatives acylated at position 4 on the quinic acid (e.g., 3,4-diCQA and 4,5-diCQA), as it has been noted in the work done by Clifford et al. [3, 10]. However, to distinguish between 3,4-diCQA and 4,5-diCQA an ion at *m/z* 335 is noteworthy, as its presence signifies the formation of a lactone group between a carboxylic group at position 1 and a hydroxyl group at position 5 on the quinic acid [27]. Thus, position 5 on the quinic acid need not be acylated to allow the formation of the lactone group as shown elsewhere [27]. Therefore, 3,4-diCQA and 4,5-diCQA can be distinguished based on the presence of the ion at *m/z* 335 on the MS^2^ spectra representing 3,4-diCQA [10].

The two peaks (Rt = 4.87 and 7.30) detected in the *M. oleifera* plant extracts were tentatively characterized as 3-caffeoylquinic acid (CQA) glycoside and 4-CQA glycoside (Table 2) [26]. The peak (Rt = 4.87) representing 3-CQA glycoside showed fragment ions (Figure 3(d)) at *m/z* 353 ([caffeoylquinic acid-H]^−^) due to the neutral loss of a glycoside residue (162 Da), 341 ([caffeoyl glycoside-H]^−^) as a result of a loss of a quinic acid moiety, 191 ([quinic acid-H]^−^) ascribed to the loss of a caffeoyl glycoside moiety, and 179 ([caffeic acid-H]^−^) (bp) due to the loss of the quinic acid and glycosyl moieties from the caffeoylquinic acid and caffeoyl glycoside groups, respectively. The peak (Rt = 7.30) representing 4-CQA glycoside showed product ions (Figure 3(e)) at *m/z* 353 ([caffeoylquinic acid-H]^−^), at *m/z* 341 ([caffeoyl glycoside-H]^−^), at *m/z* 191 ([quinic acid-H]^−^), at *m/z* 179 ([caffeic acid-H]^−^), and ion at *m/z* 173 ([quinic acid-H_2_O-H]^−^) (bp) due to the dehydration of a quinic acid moiety. The presence and abundance of the peak at *m/z* 173 allowed characterization of the peak with the Rt of 7.30 as 4-CQA glycoside [10]. For both the peaks, the presence of the ion at *m/z* 353 and 341 suggests that the caffeoyl moiety forms an ester bond with the quinic acid (to produce an ion at *m/z* 353) and an ether bond with the glucose group (to produce an ion at *m/z* 341). However, where the glycosyl group is connected on the caffeic acid catechol group (C-3′ or C-4′) is not clear. According to Jaiswal et al., an intense ion (bp) at *m/z* 323 ([caffeoyl glycoside- H_2_O-H]^−^), due to the dehydration of a caffeoyl glycoside moiety, on the MS^2^ spectra, suggests connectivity at C-3′ and an intense ion (bp) at *m/z* 353 on the MS^2^ spectra is characteristic of connectivity at C-4′ [13, 14]. In this study, both ions were secondary ions and not base peaks, and the intensity of both peaks could have been influenced by the differences in the MS conditions/parameters [28]. Thus, due to the low abundance/absence of the ion at *m/z* 323 on the MS^2^ spectra (Figures 3(d) and 3(e)), the CQA glycosides were putatively annotated as 3-*O*-(4′-*O*-caffeoyl glucosyl) quinic acid and 4-*O*-(4′-*O*-caffeoyl glucosyl) quinic acid (Figures 3(d) and 3(e); Table 2).

### 3.3. Synchronized Survey Scan (SSS)

From the above, it is apparent that in the absence of the triggered product ion scan (PIS), the optimized MRM method would have detected the diCQA and the CQA glycosides as these compounds share similar transitions. However, the method would fail to differentiate between the structurally similar compounds and thus lead to misidentification. Thus, the novel approach, SSS, allowed the detection and differentiation of all five diCQA positional isomers (Figure 2) as well as two CQA glycoside positional isomers (Figures 3(d) and 3(e)) in a single chromatographic analysis. Furthermore, the method allowed the discrimination of isobaric compounds such as diCQA and CQA glycosides. Here, an ion at *m/z* 341 was found to be present on the PIS MS/MS spectra of CQA glycosides. This ion represents caffeoyl glycoside [11] and, as seen from our results, it was only observed on the PIS spectra but not the MRM spectra. This is an indication that SSS dependent PIS allows other diagnostic ions to be used for the differentiation of closely related molecules, which otherwise would be impossible if only MRM transitions are relied upon. Thus, SSS produces an automatically performed MS/MS analysis which allows the simultaneous identification of these isomeric and isobaric compounds, without any ambiguity.

## 4. Conclusion

For the first time, we have successfully demonstrated synchronized survey scan (SSS) to be an efficient approach to detect and discriminate isomeric and isobaric plant metabolites. Briefly, this approach allowed the discrimination and identification of (1) diCQA positional isomers, (2) CQA glycosides positional isomers, and (3) isobaric compounds, diCQAs and CQA glycosides, in a single chromatographic run, albeit the developed MRM transition parameters were automatically optimized using the positional isomer 3,5-diCQA. Our results show that this method can be further applied in any method where isomers are expected. Furthermore, in phytochemistry, where identification is the key, SSS is expected to add a novel orthogonal feature during identification.

## Figures and Tables

**Table 1:** MRM transitions automatically optimized using 3,5-diCQA.

Precursor (*m/z*)	Transitions (*m/z*)	Collision energy (eV)
515	353	18
515	191	40
515	179	28

**Table 2:** MRM transitions and MRM-dependent product ion scan of isomeric and isobaric compounds from different sample types.

Sample type	Compound	Retention time (Rt) (min)	Precursor (*m/z*)	Optimal transitions	Collision energy (eV)	MRM-dependent product ion
Standard	1,3-*O*-dicaffeoylquinic acid	13.71	515	515 > 353	18	515→353, 335, 191 (bp), 179, 161, 135
				515 > 179/191	28/40	
	3,4-*O*-dicaffeoylquinic acid	21.34	515	515 > 179/353	28/18	515→353, 335, 191, 179, 173 (bp), 161, 135
				515 > 191	40	
	3,5-*O*-dicaffeoylquinic acid	22.25	515	515 > 353	18	515→353, 191 (bp), 179, 135
				515 > 191/179	40/28	
	1,5-*O*-dicaffeoylquinic acid	22.82	515	515 > 191	40	515→353, 191 (bp)
				515 > 353	18	
	4,5-*O*-dicaffeoylquinic acid	24.75	515	515 > 353	18	515→353 (bp), 191, 179, 173, 135
				515 > 179	28	
*Bidens pilosa*	3,4-*O*-dicaffeoylquinic acid	21.24	515	515 > 179/353	28/18	515→353, 335, 191, 179, 173 (bp), 161, 135
				515 > 191	40	
	3,5-*O*-dicaffeoylquinic acid	22.05	515	515 > 353	18	515→353, 191 (bp), 179, 135
				515 > 191/179	40/28	
	4,5-*O*-dicaffeoylquinic acid	24.61	515	515 > 353	18	515→353 (bp), 191, 179, 173, 135
				515 > 179	28	
*Moringa oleifera*	3-*O*-(4′-*O*-caffeoyl glucosyl) quinic acid	4.89	515	515 > 179	28	515→353, 341, 191, 179 (bp)
				—	—	
	4-*O*-(4′-*O*-caffeoyl glucosyl) quinic acid	7.32	515	515 > 179	28	515→353, 341, 191, 179, 173 (bp)
				515 > 353	18	

## Data Availability

The data used to support the findings of this study are available from the corresponding author upon request.
